# A longitudinal study of empathy in pre-clinical and clinical medical students and clinical supervisors

**DOI:** 10.1186/s12909-016-0777-z

**Published:** 2016-10-18

**Authors:** Sarah Mahoney, Ruth M. Sladek, Tim Neild

**Affiliations:** Flinders University, PO Box 494 Noarlunga Centre, Adelaide, South Australia 5168 Australia

**Keywords:** Empathy, Medical education, Medical students, Clinical supervisors, Longitudinal integrated clerkships

## Abstract

**Background:**

Although appropriate empathy in health professionals is essential, a loss of empathy can occur during medical education. The structure of clinical learning may be one factor that is implicated in a loss of empathy. This study examines student and doctor empathy, and possible associations between empathy and the structure of clinical learning.

**Methods:**

There were three groups of participants: medical students (*n* = 281), who completed a longitudinal survey consisting of the Jefferson Scale of Empathy and an open question about empathy at the beginning and end of the 2013 academic year; private doctors (medical practitioners) in South Australia (*n* = 78) who completed a survey consisting of the Jefferson Scale of Empathy and an open question about empathy at the end of the students’ academic year; and doctors (medical practitioners) from public teaching hospitals (*n* = 72) in southern Adelaide, South Australia who completed a survey consisting of the Jefferson Scale of Empathy at the end of the students’ academic year .

**Results:**

Year one students’ empathy scores at the end of the year (102.8 ± 17.7) were significantly lower than at the start of the year (112.3 ± 9.6) *p* < .05). There were no other significant differences in students’ empathy scores by year groups or across the two time points. Empathy scores were almost identical for private and hospital clinicians and higher than average scores for students. Free-text comments highlighted the importance students and doctors place on empathy. Students described issues that adversely affected their empathy, including specific incidents, systemic issues, and course structure, but also described some positive role models. Doctors’ comments focused on the importance of empathy but qualified its meaning in the therapeutic setting.

**Conclusion:**

Medical students and practitioners alike ascribe importance to empathy in clinical practice, yet its developmental course remains poorly understood with possible decrement across the course of medical education. A more sophisticated understanding of empathy in medical students is needed, with attention to issues that might adversely impact on this crucial aspect of their development.

**Trial registration:**

This was not undertaken as the research did not involve a health care intervention on human participants.

**Electronic supplementary material:**

The online version of this article (doi:10.1186/s12909-016-0777-z) contains supplementary material, which is available to authorized users.

## Background

The importance of appropriate empathy in health professionals is well recognised, and empathy is highly valued by the community as a component of the doctor-patient interaction. It is also of major importance to students and to the profession, as loss of empathy can be associated with lower professional satisfaction and burnout [1], and may adversely impact on professional standards. A loss of empathy by medical students as they progress through their studies has been reported by authors in a number of countries, occurring particularly in the clinical years of the course. However the evidence is mixed; for example, schools in Japan [2], Portugal [3], Australia [4] and United Kingdom [5] have demonstrated little or no loss of empathy in their students, while studies in Iran [6], New Zealand [7], and North America (Florida, Boston [8]; Philadelphia [9]) have shown it to be a common issue. A systematic review by Neumann et al. in 2011 [10] concluded (from mostly American data) that empathy decline during medical school and residency compromises development of professionalism and may threaten health care quality.

Early findings in longitudinal clinical training programs [11] (also called longitudinal integrated clerkships or LICs), where students spend at least 6 months with the same clinical supervisor and have the ability to see the same patients over a period of time, suggest that this type of program structure for clinical learning may decrease the trend to lower empathy scores [12]. These findings are however yet to be clearly replicated. Furthermore, LICs in different institutions, or even in the same institution, are not identical, and it is difficult to know which factors might be influencing the outcomes. In addition to program structure, clinical learning environment, student selection, and cultural influences may all play a part, as well as student maturity, age, gender, and background. More recently some authors have questioned how empathy should be measured, and whether erosion of empathy really occurs [13].

This research aimed to examine the associations between empathy and gender, age, previous study or career, stage of medical studies, structure of early clinical learning program, and empathy scores of clinical supervisors for medical students in an Australian medical school. Medical students in the Flinders Doctor of Medicine (MD) program are graduate entry students who undertake a 4-year program. In years 1 and 2 all students study on campus, predominantly learning clinical skills and the biomedical sciences. Year 3 students are immersed in the clinical workplace in a variety of geographically diverse year-long programs. Approximately half of the year 3 student group undertake traditional tertiary hospital block rotations at Flinders Medical Centre (FMC). The other half undertake alternative programs of which around one-third are in rural longitudinal integrated clerkships, one third in an urban community-based longitudinal program, and one third in a hybrid hospital / community / rural program. Year 4 students all undertake multiple 6-week rotations through a variety of hospital, rural and community placements.

## Methods

### Design

A longitudinal survey was used.

### Participants

Three groups of participants were invited. *Medical students*: All medical students enrolled in the 4-year Doctor of Medicine (MD) Course at Flinders University in South Australia in 2013. *Private Doctors:* All doctors in private practice in the rural and urban locations where students undertake community-based clinical placements. *Public Doctors:* All doctors working in the public hospitals in the Southern Adelaide Local Health Network, which includes the tertiary hospital Flinders Medical Centre.

The survey was repeated at two time points for medical students. Time point one (TP1) was the start of the 2013 academic year (January) and time point two (TP2) was in October, in order to be near the end of the 2013 academic year. A total number of 281/594 (47.3%) medical students responded at TP1; 84/594 (14.1%) at TP2, and 53/594 (8.9%) at both time points. Both public and private doctors were invited at TP2 only (Additional file 1). Response rates are estimated at 13.0% (78/600) and 9.1% (73/800) respectively as the exact number of doctors at the time of invitation is unknown.

### Procedures

Ethics approval was provided by the Flinders Behavioural Research and Ethics Committee (project ID: 5807 for medical student survey, and ID 5937 for private doctor survey) and the Southern Adelaide Clinical Human Research Ethics Committee (project ID: 425-12) for the public doctor survey.

Commencing year 1 students were invited once to participate during an introductory lecture session at TP1. All further student invitations (subsequent year 1 TP1, year 1 TP2 and both time points for students in years 2-4) were made via generic emails through the whole of class electronic communication system known as Flinders Learning Online. Two repeat generic emailed invitations were sent to all students in all year levels at two weeks and four weeks after the initial invitation.

Private doctors were identified through entries in the South Australian telephone directory ‘Yellow Pages’ [14] for each of the regions where Flinders medical students had clinical placements. They were invited by email where an email address was available, and otherwise by a posted invitation and hard copy survey. Public doctors were invited by email through their employer, the Southern Adelaide Local Health Network (SALHN). The survey was made available to all participants in two formats, either electronically through SurveyGizmo© [15] or in hard copy via a printed questionnaire booklet. Two follow up emails were provided to all participants initially invited by email.

While the participant groups to be contacted were chosen purposively, the constraints imposed by the ethics committees effectively resulted in a volunteer sampling outcome.

### Measures

#### Background

The student version of the survey required participants to self-allocate a User ID code to enable surveys completed at both time points to be matched correctly while ensuring de-identification of participants. Demographic information and characteristics collected included age, gender, previous undergraduate degree, previous health professional qualifications, previous years of clinical work experience and medical specialty aspirations post-MD graduation. The doctor version of the survey asked for seniority, specialty and the extent and site of teaching activities, but was otherwise limited. This was to ensure a doctor’s anonymity was preserved given that additionally knowing age and gender might otherwise have revealed their identity in a relatively small professional community.

#### Empathy

Empathy was measured using Student and Physicians’ respective versions of the Jefferson Scale of Empathy (JSE), the most widely used and validated measure of empathy [16, 17]. The JSE is a validated, 20-item scale designed to measure empathy in health care practitioners and students. Student and physician versions of the JSE are similar in content with minor modifications in wording of some items to maintain face and content validities for the different target populations [18]. The current sample provided internal reliabilities (Cronbach’s alpha) of 0.78 (Students TP1), 0.85 (Students TP2), 0.79 (Public Doctors) and 0.69 (Private Doctors).

#### Personal experiences

The final question of the survey was either ‘In this section, please comment as you wish on any matters relating to empathy and/or to ethical behaviour as it relates to your role as a medical student’ (for medical students) or ‘In this section, please comment as you wish on any matters relating to empathy and/or to ethical behaviour as it relates to your role as a practising clinician’ (for private doctors). This question was approved by the University ethics committee and included in all student surveys and the private doctors’ survey. However permission to survey doctors working in the publicly funded service also required approval by the public health service’s ethics committee which refused permission to include this question. Although it was not possible to use this question for the public doctors group of participants, the researchers felt that valuable information could still be obtained from the other participant groups and so it remained in the surveys used for students and private doctors.

### Statistical analyses

Data were coded into IBM SPSS [19]. Paired t-tests, independent t-tests and ANOVAs were used as described in the text and tables.

Only *n* = 10 students from four different Year 3 program sites responded at both time points, providing too few participants to warrant a planned statistical analysis of between or within group differences across the two time points by clinical instruction site. An unplanned analysis was completed to explore difference in empathy scores in two ways: (1) by stage of clinical immersion (Stage 1 = students at the start of year 3 just prior to their first clinical year; Stage 2 = comprising year 3 TP2 and year 4 TP1) and (2) by year 3 clinical instruction site (FMC Traditional Rotations compared with all other sites). Results are reported in the Results section under ‘Year 3 site’.

## Results

### Students

Table 1 shows the characteristics of students who completed the survey at either TP1 only or at both time points, and Table 2 provides empathy scores for pre-clinical and clinical students.

**Table 1:** Characteristics of medical students who completed at both time points one *and* two* (TP1 & 2) and time point one only (TP1)

	Year 1 (*n* = 140)		Year 2 (*n* = 52)		Year 3 (*n* = 43)		Year 4 (*n* = 46)	
	Completed TP1 & 2 (*n* = 15)	Completed** TP1 (*n* = 125)	Completed TP1 & 2 (*n* = 16)	Completed TP1 (*n* = 36)	Completed TP1 & 2 (*n* = 10)	Completed TP1 (*n* = 33)	Completed TP1 & 2 (*n* = 12)	Completed TP1 (*n* = 34)
Age at TP1 (years)	26.7 ± 6.6	24.1 ± 4.2	30.1 ± 7.1	25.8 ± 4.7	32.1 ± 7.5	27.5 ± 5.7	27.5 ± 5.2	28.1 ± 5.6
Gender (female, %)	9 (60.0 %)	68 (55.3 %)	11 (68.8 %)	17 (47.2 %)	7 (70.0 %)	21 (63.6 %)	8 (66.7 %)	22 (64.7 %)
Year 3 Site								
FMC Traditional blocks	-	-	-	-	3 (30.0 %)	5 (15.2 %)	2 (16.7 %)	11 (32.4 %)
Alternative year 3 programs	-	-	-	-	7 (70 %)	28 (84.8 %)	10 (83.3 %)	23 (67.6 %)
Number (%) of students with previous clinical experience	4 (26.7 %)	24 (19.2 %)	6 (37.5 %)	12 (33.3 %)	4 (40.0 %)	6 (18.2 %)	5 (41.7 %)	9 (25.7 %)
Empathy								
Time 1 JSE	112.3 ± 9.6^b^	110.8 ± 10.8	115.6 ± 7.9	115.2 ± 9.3	116.3 ± 12.1	115.6 ± 10.3	114.4 ± 8.4	113.9 ± 10.5
Time 2 JSE	102.8 ± 17.7^a,b^	-	115.7 ± 11.0^a^	-	111.1 ± 6.5	-	112.2 ± 9.2	-

*A small number of students who completed the survey at time point 2 only are not included in this table

**Note: Each column headed ‘completed TP1’ excludes any participants who participated at *both* TP1 and TP2 (which are specified in the alternate column/s)

^a-b^ *p* < .05

**Table 2:** Clinical and pre-clinical student JSE scores (paired t-tests)

	*n*	JSE (Mean ± SD)	*p*
TP1			
Pre-Clinical students^a^	235	112.8 ± 10.5	.48
Clinical students^b^	46	114.0 ± 9.9	
TP2			
Pre-Clinical students^c^	46	110.4 ± 15.9	.73
Clinical students^d^	38	111.4 ± 7.5	

^a^Pre-clinical students include students in Year 1 (*n* = 140), Year 2 (*n* = 52), and Year 3 TP1 (*n* = 43)

^b^Clinical students include students in Year 4 (*n* = 46)

^c^Pre-clinical students include students in Year 1 (*n* = 23) and Year 2 (*n* = 23)

^d^Clinical students include students in Year 3 TP2 (*n* = 25) and Year 4 (*n* = 13)

#### Empathy

An ANOVA found significant group differences between course years for those who only participated at TP1 (See Empathy in Table 1, columns 2, 4, 6 and 8; F (3, 224) = 3.010, *p* = .031). However Bonferroni Post Hoc tests failed to find any significant pairwise differences at *p* < .05. A further ANOVA was used to investigate potential between-group differences for all those who participated at both TP1 and 2 (See Empathy in Table 1, columns 1, 3, 5 and 7). A statistically significant difference was found for TP2 only ((F (3, 49) = 2.968, *p* = .041). Post hoc comparisons using the Bonferroni test confirmed that mean score for Year 1 (102.8 ± 17.7) was significantly lower than for Year 2 (115.7 ± 11.1, *p* < .05).

Within-year group differences between the two time points were examined using paired t-tests. Only one significant difference was identified. Empathy scores for Year 1 students decreased from 112.3 ± 9.6 at TP1 to 102.8 ± 17.7 at TP2 (*t* (14) = 2.2, *p* = .045) (See Empathy, Table 1, Column 1).

When TP1 participants were allocated into either a pre-clinical and clinical group, independent t-tests failed to find any significant differences in empathy scores comparing students in their pre-clinical years (years 1, 2 or 3) and those with at least one year of clinical immersion (year 4) at TP1. At TP2 all year 3 respondents were grouped with year 4 students as they were at the end of at least one year of clinical immersion. Similarly, no between-group differences in empathy scores were identified (Table 2).

#### Year 3 Site (Table 3)

**Table 3:** Empathy scores (JSE) for stage of clinical immersion by year 3 clinical site for students who have only completed 1 clinical year (Mean ± SD)

	Year 3 Clinical Site (*n* = 114)		
	FMC Traditional (*n* = 30)	Other (*n* = 84)	Comparison by site
	Mean ± SD	Mean ± SD	*p* value
Stage			
Stage 1 (*n* = 43) (Start of year 3^a^)	107.3 ± 9.2 (*n* = 8)	117.7 ± 10.0 (*n* = 35)	.01
Stage 2 (*n* = 71) (= one year of clinical immersion^b^)	113.6 ± 9.3 (*n* = 22)	112.5 ± 9.0 (*n* = 49)	.63
Comparison by Stage *p* value	.10	.01	

^a^TP1 Year 3 students (*n* = 43)

^b^TP1 Year 4 students (*n* = 46); TP2 Year 3 students (*n* = 15); TP1 Year 3 students who also responded at TP2 (*n* = 10)

Amongst Stage 1 students (just prior to commencing their first clinical year), those undertaking a FMC Traditional Rotation had a lower average empathy score (107.3 ± 9.2) than the combined group of all other students at other sites (117.7 ± 10.0, *t*(41) = 2.71, *p* = .01). In comparison, amongst Stage 2 students (those who had completed one clinical year), there were no significant differences in empathy scores between FMC Traditional Rotations students and all other students.

Amongst FMC Traditional Rotations students, there were no significant differences between Stage 1 and Stage 2 students, however there was a trend towards higher empathy scores in Stage 2 students (113.6 ± 9.3) compared with Stage 1 (107.3 ± 9.2, *t*(28) = 1.67, *p* = .106). In comparison, amongst all other students at other sites, students at Stage 2 (112.5 ± 9.0) had a significantly lower mean empathy scores than their Stage 1 counterparts (117.7 ± 10.0, *t*(82) = 2.51, *p* = .01).

#### Gender

There were no significant gender differences in empathy scores amongst year 2 or year 3 respondents at either time point. However female year 1 students (114.1 ± 8.7) had significantly higher empathy scores at TP1 than males (107.1 ± 11.8, *t*(136) = 3.976, *p* < .001). This was also true at TP2, with year 1 females scoring significantly higher (113.2 ± 8.9) than males (91.9 ± 24.3), *t*(21) = 3.073, *p* = .006). For year 3 students at TP2, females scored significantly higher (114.3 ± 4.1) than males (106.3 ± 7.1, *t*(23) = 3.57, *p* = .002.

### Doctors

Table 4 shows the baseline characteristics of responding doctors. Public doctors came from a variety of disciplines such as anaesthesia, emergency medicine, internal medicine, obstetrics and gynaecology, paediatrics, pathology, psychiatry, and surgery but excluded family medicine. Amongst private doctors, 70.5% were family medicine specialists (also known as general practitioners) and 29.5% were specialists across a similar wide range of specialties as in the public system. In both settings almost all of the respondents were senior practitioners with eight or more years post-specialty experience. Most of the public doctors (87.5%) and more than half (52.6%) of the private doctor respondents reported teaching medical students. In both groups most respondents taught a range of other trainees including those in their own and other specialisations, and both students and trainees in other health professions.

**Table 4:** Characteristics of Doctors (*n* = 150)

	Public	Private
	*n* = 72	*n* = 78
JSE Mean ± SD	116.9 ± 9.9	115.6 ± 9.4
Years since graduation		
≤7 years	4(5.6 %)	1 (1.3 %)
≥ 8 years	68 (94.4 %)	77 (98.7 %)
Seniority		
Pre-vocational	6 (8.3 %)	1 (1.3 %)
Specialist qualification	66 (91.7 %)	77 (98.7 %)
Work location		
Tertiary Hospital	66 (91.7 %)	9 (11.5 %)
Urban Private Practice	-	64 (82.1 %)
Rural Private Practice	-	4 (5.1 %)
Other	6 (8.3 %)	1 (1.3 %)
Teaching experience^a^		
Not teaching	1 (1.4 %)	16 (20.5 %)
Teaching medical students	63 (87.5 %)	41 (52.6 %)
Speciality		
General Practice (Family Medicine)	-	55 (70.5 %)
All other specialties	72 (100 %)	23 (29.5 %)

^a^Some respondents teach postgraduate trainees or trainees from other health professions

Independent t-tests failed to find any significant differences in empathy scores between public and private doctors, nor any differences by work location, specialty or teaching experience. When empathy scores between public doctors, private doctors and all TP2 student respondents were compared using an ANOVA, no significant differences were found.

### Thematic analysis

Students and private doctors were asked to provide free-text responses to either: ‘In this section, please comment as you wish on any matters relating to empathy and/or to ethical behaviour as it relates to you role as a medical student’ or ‘In this section, please comment as you wish on any matters relating to empathy and/or to ethical behaviour as it relates to your role as a practising clinician’.

Responses to the free text question were analysed using an inductive content analysis and open coding approach [20] to determine common themes. These are reported descriptively. Of the 388 students who completed the survey at any time point, 159 (41.0%) gave free text comments. The three major themes of these comments were:

1. Empathy is an important quality for doctors and the doctor-patient relationship, and improves standards of care. For example: ‘*… understanding the perspective of other people is key to forming a strong rapport and building trust …*’ ID 86;
2. A lack of empathy in the clinical workplace or medical school had been observed, for example: *‘I have found that … empathy for the patient gets pushed to one side and learning becomes the main priority*’ ID 66;
3. Medical education can decrease empathy, as can systemic issues such as workload, or service structure e.g. shift rotations can decrease empathy. Such issues create fear of burnout and need for self-protection, for example: *‘The structure of medical education tends to drain empathy from the students. We are constantly being told we need to be empathetic while being shown no empathy ourselves…’* ID 16.

Other issues identified as adversely affecting empathy included: empathy being actively discouraged, clinical team behaviours and systemic or service issues, need for self-protection, frustration with student role, uncertainty about the meaning of empathy and the ability to develop it, and a need to balance empathy with objectivity. *‘…I think that there is a fine line between being empathetic enough to gain the trust of patients, and then becoming too detrimentally empathetic …’* ID 91. *‘…At times it can be difficult to separate empathy from sympathy, and in sympathy there is less objectivity…’* ID 31.

A small number of students reported observing good role models, for example: *‘…after being exposed to other doctors who have exceptional skills in relating to their patients, I have become much less disenchanted*…’ ID 118.

A similar analysis of the private doctors’ free-text responses was done. Comments were made by 41 (53%) of private doctor respondents. The two dominant themes were:

1. Empathy is essential to the therapeutic relationship.
2. Qualifying statements about empathy and the therapeutic relationship: Some said that more than empathy is required: *‘patients want … their physician to be understanding and empathetic of their situation and yet still clear-headed and objective in their analysis and management’* ID 7; others said that the patient-doctor relationship requires boundaries ‘…*Need some level of detachment and professionalism because patients' views often unrealistic, emotive, influenced by bias (es)…*’ ID17. Empathy was seen by some to confuse the doctor-patient relationship and affect outcomes, and as being difficult to define. ‘*Doctors’ mode of communication differs radically from 'normal' communication…’ ID 64*; and some thought that doctors’ health and working conditions affect empathy. Some had seen instances of poor empathy by other doctors. Some respondents specifically commented on empathy and medical education: *‘the medical school experience is very damaging to the emotional development of future doctors’* ID 54, *‘…very important to also have empathy for students - what they are going through and their issues…*’ ID 50.

## Discussion

The results of this research showed a decrease in empathy scores for Year 1 students across an academic year, but limited other significant differences between year groups or across the time points. Average empathy scores for students were consistent in range with those from other studies, and are comparable to the mean JSE scores of 115 for students and 120 for clinicians gathered by Hojat Gonnella and Maxwell [21]. In particular there was no overall decline in empathy during the clinical years, consistent with other Australian research [4]. As with previous research, females were more empathetic than males. A power calculation was not undertaken *a–priori* given that all students were targeted.

Most Year 1 students completed the survey during their first lecture, arguably the time when a medical student has the most heightened ideals of a career in medicine. The subsequent decrease in empathy by the end of Year 1 may reflect this, but also supports prior research which has increasingly proposed that empathy is a state as opposed to a trait, and is therefore modifiable and educable [22].

Flinders Medical School provides year 3 students with multiple site options for year 3 which is their first year of clinical immersion. Students spend a full year in the site of their choice, and depending on the site, programs are structured either as a rotations-based program, a rural longitudinal integrated clerkship (LIC), or a hybrid community LIC. Our interest in the potential change in empathy in relation to the site of clinical instruction at Year 3 could not be evaluated statistically given the low response rate. We therefore combined the results from students at the end of year 3 and those starting year 4, since both these groups had effectively completed one year of clinical immersion. Analysis using the combined data allowed some statistically significant differences to be seen. Results of this indicate that there is no overall decline in empathy in the clinical years of the course, but suggests that students opting for non-rotations programs start their clinical year with a higher average JSE than those choosing the tertiary hospital rotations program. This could mean either that students with higher starting empathy scores self-select to non-rotations programs, or the converse, that those with lower JSE scores choose the rotations program. At the end of the year, whatever clinical training program was undertaken in year 3, students’ empathy scores at the end of the first clinical year were very similar and did not demonstrate any overall decline in empathy.

The average empathy scores of clinician respondents were the same for private and hospital clinicians and higher than average overall scores for students. In general, most of the hospital doctors who teach are involved with rotations-based year 3 programs, while the LICs and hybrid programs predominantly engage private clinicians as supervisors. As role-modelling is an important factor in empathy development and maintenance for students [23], the consistency of empathy scores for a sample of clinical supervisors in various settings is reassuring for program designers. The consistency of supervisor empathy scores may also correlate with the consistency and similarity of empathy scores for students. It is important to note however that almost all clinician respondents were senior practitioners at specialist level with at least 8 years of clinical experience. In the public hospital system in particular, students are also potentially influenced by junior trainee doctors who contribute significantly to patient care in such environments. Further research is needed to clarify whether differences in empathy exist between junior and senior doctors in Australian hospitals.

In their free-text comments, students and doctors in private practice highlighted the importance of empathy. Both groups showed some uncertainty about the meaning of empathy and the need to balance empathy with objectivity, and many recognised the imperative for self-care, seeing this as a barrier to empathy. Students made comments about adverse events or difficulties in understanding and developing empathy, suggesting that more needs to be done to help students with this crucial aspect of their development as clinicians. Medical students, particularly in the clinical years, spend much of their time in the workplace where the personalities and behaviours of workers, as well as organisational systems and structures, will all impact on any individual student’s experiences [24, 25]. While a few students gave examples of positive experiences, most of the student comments were negative. However, given the overall low response rate and volunteer sampling method, it is possible that those students who had significant concerns may have been more likely to respond to the survey in general and this question in particular than those who did not have any concerns.

Doctors’ free text responses reflected their experiences of empathy and professionalism in clinical practice. They described empathy as essential to the therapeutic relationship while also acknowledging that the patient-doctor relationship requires boundaries, reflecting the different interpretation by clinicians of the meaning of empathy as discussed by Halpern [26]. The qualifications on the meaning of empathy provided by many of the respondents are consistent with the ‘…tension in medical professionalism between the image of the clinically competent doctor and the caring doctor’ discussed by Kerasidou and Horn [27] who also highlight that ‘Empathy is an important aspect of clinical care, but the emotional labour it requires is not negligible.’ This research shows a lack of clear differences in empathy scores between various groups of students, and between students and clinicians, and provides some support for Roff’s call [13] for a more sophisticated understanding of empathy in health professionals. Perhaps we should also be striving to look for changes in the systemic and structural issues [22] that contribute to emotional distress [28] in medical students and trainees, and work to improve those rather than only focussing on the student side of the equation. Limitations of this research are predominantly due to a disappointing response rate whereby, although participant groups to be contacted were chosen purposively, constraints imposed on the research effectively resulted in a volunteer sampling outcome with consequent low responses compared to the total potential cohort available. As a result it is possible that the data has a self-selection bias in both students and clinicians. Furthermore we received very few responses from junior doctors, who are important contributors to the learning and clinical environment particularly in the public hospital system. Another limitation is that we have only the student or clinician voice and do not have the patient voice.

## Conclusion

This study contributes further empirical data to the question of empathy among medical students and practising doctors in Australia. However its developmental course across medical education requires further research. Further, this study demonstrated that both students and clinicians attach importance to empathy in professional practice. As schools like Flinders Medical School consider restructuring clinical experiences into longitudinal clerkships (and other models), it is imperative that assumptions about the development, protection and maintenance of empathy are further explored and that the relationship between the clinical learning environment and empathy is better understood. If the structure of clinical experiences can impact what is widely acknowledged to be critical to patient-doctor interactions, medical educationalists have a responsibility to understand this relationship.

## Additional file

Additional file 1: Flow chart of sampling process. (DOCX 28 kb)
