# Supplementary material for: A longitudinal study of empathy in pre-clinical and clinical medical students and clinical supervisors
Source: BMC Med Educ. 2016 Oct 18;16:270. doi: 10.1186/s12909-016-0777-z (PMC5070083; doi:10.1186/s12909-016-0777-z)
Supplement: Additional file 1: — Flow chart of sampling process. (DOCX 28 kb) [file 12909_2016_777_MOESM1_ESM.docx]

Private and public doctors offered survey once only

All year 4 students offered 2^nd^ survey

All year 4 students offered 1^st^ survey

All year 3 students offered 2^nd^ survey

All year 3 students offered 1^st^ survey

All year 2 students offered 2nd survey

All year 2 students offered 1^st^ survey

All year 1 students offered 2^nd^ survey

All year 1 students offered 1^st^ survey

Time Point 2 October 2013

Time point 1 January 2013

January 2013

Survey period January – October 2013
